# A 2-Question Summative Score Correlates with the Maslach Burnout Inventory

**DOI:** 10.5811/westjem.2020.2.45139

**Published:** 2020-04-21

**Authors:** Simiao Li-Sauerwine, Katie Rebillot, Matthew Melamed, Newton Addo, Michelle Lin

**Affiliations:** * The Ohio State University, Department of Emergency Medicine, Columbus, Ohio; † Los Angeles County + University of Southern California, Department of Emergency Medicine, Los Angeles, California; ‡ New York Presbyterian Brooklyn Methodist Hospital, Department of Emergency Medicine, Brooklyn, New York; § University of California, San Francisco, Department of Emergency Medicine, San Francisco, California

## Abstract

**Introduction:**

There is a high prevalence of burnout among emergency medicine (EM) residents. The Maslach Burnout Inventory - Human Services Survey (MBI-HSS) is a widely used tool to measure burnout. The objective of this study was to compare the MBI-HSS and a two-question tool to determine burnout in the EM resident population.

**Methods:**

Based on data from the 2017 National Emergency Medicine Resident Wellness Survey study, we determined the correlation between two single-item questions with their respective MBI subscales and the full MBI-HSS. We then compared a 2-Question Summative Score to the full MBI-HSS with respect to primary, more restrictive, and more inclusive definitions of burnout previously reported in the literature.

**Results:**

Of 1,522 residents who completed the survey 37.0% reported “I feel burned out from my work,” and 47.1% reported “I have become more callous toward people since I took this job” once a week or more (each item >3 on a scale of 0–6). A 2-Question Summative Score totaling >3 correlated most closely with the primary definition of burnout (Spearman’s rho 0.65 [95% confidence interval 0.62–0.68]). Using the summative score, 77.7% of residents were identified as burned out, compared to 76.1% using the full MBI-HSS, with a sensitivity and specificity of 93.6% and 73.0%, respectively.

**Conclusion:**

An abbreviated 2-Question Summative Score correlates well with the full MBI-HSS tool in assessing EM resident physician burnout and could be considered a rapid screening tool to identify at-risk residents experiencing burnout.

## INTRODUCTION

### Background

Physician burnout is a well-described problem that has been demonstrated to impact physician performance, patient care, and institutional expenditure, and begins in training as early as intern year.^1^,^2^ The narrative definition of burnout is a complex, multidimensional, psychological syndrome resulting from long-term stress during one’s career.^3^,^4^ The World Health Organization defines burnout as an occupational phenomenon based on the *International Classification of Diseases*, 11^th^ revision (ICD-11), which states that burnout is “a syndrome conceptualized as resulting from chronic workplace stress that has not been successfully managed” and includes the three dimensions of feeling “energy depletion or exhaustion; increased mental distance from one’s job, or feelings of negativism or cynicism related to one’s job; and reduced professional efficacy.”^5^ Because of its significant impact on various facets of healthcare delivery, much interest has been dedicated to the best means to quantify burnout, in order to develop a meaningful measure to address its prevalence and the impact of interventions to reduce burnout.

The Maslach Burnout Inventory-Human Services Survey (MBI-HSS) is a widely used tool to measure burnout and has been validated in the physician population.^6^ Its three subscale domains are emotional exhaustion (a state of emotional depletion at work [EE]), depersonalization (a lack of feelings or negative and/or cynical feelings toward others [DP]), and personal accomplishment (a sense of success at work [PA]). In interpreting the burnout scale, various definitions have been proposed, from low, primary, and high subscales for each domain to a dichotomous “burned out/not burned out” definition.

### Importance

Burnout rates are highest in the emergency physician population and burnout is broadly acknowledged to be a prevalent and significant problem with respect to physician health and impact on patient care.^6^–^8^ In a recent national cross-sectional survey of the prevalence of burnout in emergency medicine (EM) residents, three-quarters of them met criteria for burnout;^9^ this both illustrates that the EM resident population is vulnerable to the negative effects of burnout and highlights this population as one ripe for intervention. However, certain obstacles exist in studying burnout prevalence and effects of interventions in this population, chief among them the burden of administering the lengthy MBI-HSS instrument to a population stressed by limited time and competing demands.

### Goals of This Investigation

Brief measures of burnout based on the MBI-HSS have been studied in physician populations. A two-item abbreviated MBI addressing the domains of EE and DP correlates highly with the full MBI-HSS in various cohorts of medical students, non-EM residents, and practicing physicians.^10^–^12^ We aimed to validate the use of the same two-item MBI in a national cohort of EM residents in order to provide a rapid tool that may be used by researchers, residency program leadership, and EM residents themselves to assess and track burnout trends. To our knowledge, this is the first study to validate the two-item MBI in a national sample of EM residents.

## METHODS

### Survey Tool

The 2017 National EM Wellness Survey was administered by the Academic Life in Emergency Medicine (ALiEM) organization and its Wellness Think Tank volunteer initiative. ALiEM is a nonprofit, health professions education organization focused on social media technologies and community building. The Wellness Think Tank is an online community comprised of United States (US) EM residents and faculty advisors interested in physician wellness. Using the ALiEM website, social media, and listservs including those of the Council of EM Residency Directors and the EM Residents Association, we conducted our 2017 National EM Resident Wellness Survey March 20–31, 2017, focusing only on US EM residents. The survey included the full MBI-HSS questionnaire^13^ and was hosted online on REDCap version 8.1.4 (Research Electronic Data Capture, Vanderbilt University, Nashville, TN), a secure web application for building and managing online surveys and databases.^14^ The study was granted expedited review by the institutional review board of New York Presbyterian Brooklyn Methodist Hospital.

**Population Health Research Capsule**

What do we already know about this issue?

There is a high prevalence of burnout among emergency medicine (EM) residents. The Maslach Burnout Inventory (MBI) is a widely used and well-validated tool to measure burnout.

What was the research question?

Can we create a robust, rapid tool to measure burnout in EM residents?

What was the major finding of the study?

A 2-Question Summative Score >3 correlated with the MBI, with a sensitivity and specificity of 93.6% and 73.0%, respectively.

How does this improve population health?

The brief 2-Question Summative Score correlates with the MBI and can be used as a rapid screening tool to identify at-risk residents experiencing burnout.

Although physician burnout is defined in a variety of ways using the MBI-HSS tool,^6^ the commonly used definition, which we also used in our original study, was a high EE (≥27) or high DP (≥10) score. Two alternative definitions are high EE (≥27) or high DP (≥10) or low PA (≤33), which we label as ”more inclusive,” and high EE (≥27) and high DP (≥10) and low PA (≤33), which we label as “more restrictive.”^9^,^10^ Detailed methodologies on identifying, recruiting, and administering the confidential, online, full MBI-HSS survey tool can be found in the original publication.^9^ The prevalence of burnout among EM residents from the original study was 76.1% (95% confidence interval [CI], 74.0–78.3%). Using the more inclusive and more restrictive definitions, 80.9% (78.9–82.9%) and 18.2% (16.3–0.1%) of EM residents were burned out, respectively.

### Outcome Measures

Based on previously published data on 1,522 US EM residents from the 2017 National EM Wellness Survey, we assessed the performance of the validated, two-item abbreviated item MBI tool relative to the full MBI-HSS tool for measuring burnout in EM residents. Based on previous studies, the two nested questions that have demonstrated the highest factor loading for the EE and DP domains were “I feel burned out from my work” (EE1) and “I have become more callous toward people since I took this job” (DP1), respectively. Although each are scored on a seven-point Likert scale (0–6), these two items were dichotomized as burned out if respondents described a frequency of once a week or more often, based on previously reported thresholds.^10^ Thus, a score >3 for EE1 or DP1 was defined as burned out for either item.

### Data Analysis

With the main aim to assess the performance of EE1 and DP1 relative to their subscales and their association with resident burnout, we calculated the response distributions using standard descriptive statistics and evaluated the bivariate associations by calculating Spearman’s correlations between the two single-items (EE1 and DP1), their respective subscales, and each of the burnout definitions. Of note, the subscales corresponding to “emotional exhaustion” (EE) and “depersonalization” (DP) were adjusted with the two single-item questions removed and are reported as EE(−EE1) and DP(−DP1), respectively. We calculated test characteristics for a “2Q Summative Score,” which adds the EE1 and DP1 item scores. Cutoffs of EE1 >3 and DP1 >3 were used for calculating both odds ratios and classification accuracy measures (sensitivity, specificity, positive predictive value, negative predictive value) for resident burnout based on the primary, more inclusive, and more restrictive definitions.

## RESULTS

### Characteristics of Study Subjects

A total of 1522 of 7186 US EM residents (21.2%) representing 193 of 247 residency programs (78.1%) participated in the survey. Further details regarding the study population, including inverse probability weighting to adjust for non-response bias, are available in the original publication.^9^

### Main Results

**Figure 1:**
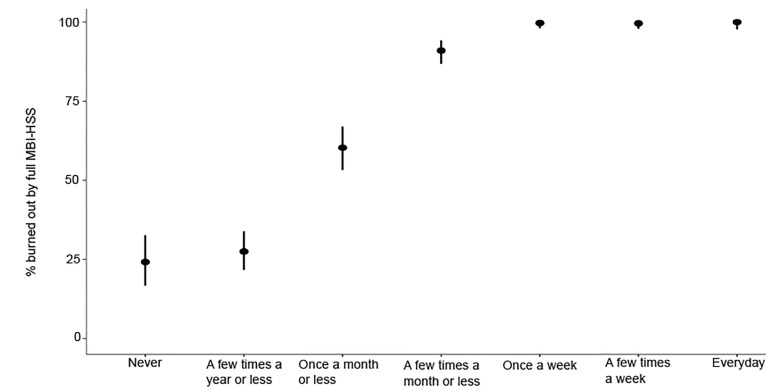
Prevalence of resident burnout stratified by emergency medicine resident response to the question “I feel burned out from my work” (EE1). *MBI-HSS*, Maslach Burnout Inventory-Human Services Survey.

**Figure 2:**
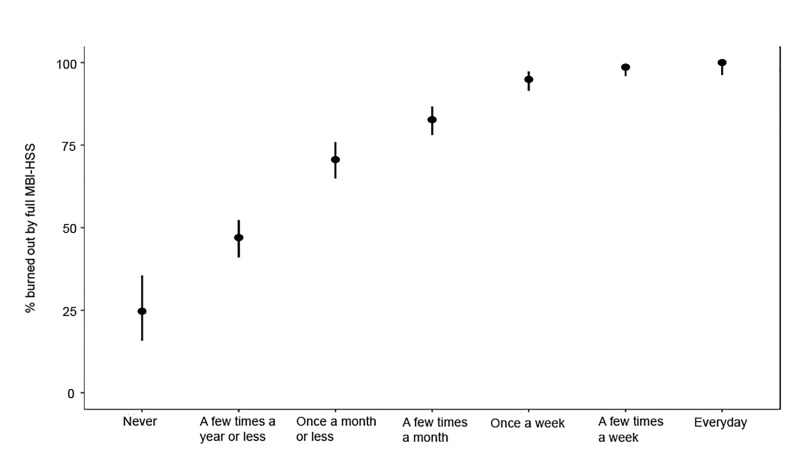
Prevalence of resident burnout stratified by emergency medicine resident response to the question “I have become more callous toward people since I took this job” (DP1). *MBI-HSS*, Maslach Burnout Inventory-Human Services Survey.

**Figure 3:**
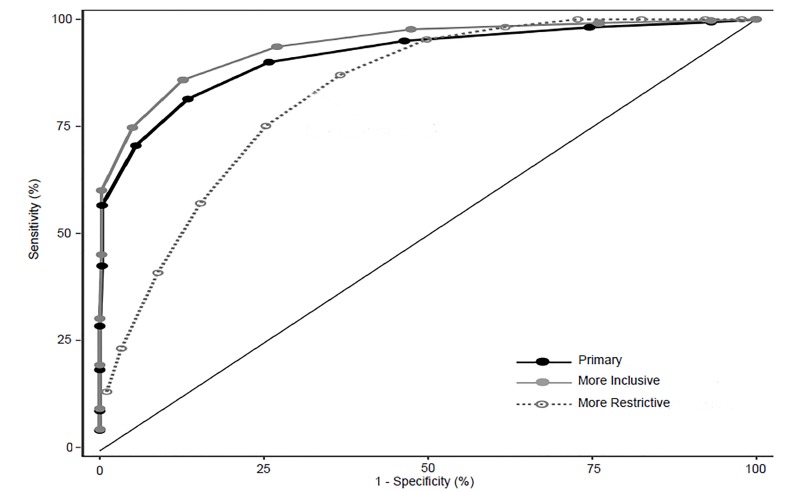
Receiver operating characteristic of primary, more inclusive, and more restrictive definitions of burnout based on the 2-Question Summative Score cutoffs. Dots represent a cutoff score of 0 to 12 from right to left on each curve.

The frequency of responses for questions EE1 and DP1 are reported in Table 1 with 37.0% and 46.8% of residents experiencing these once a week or more (score >3), respectively. The prevalence of resident burnout using the full MBI-HSS tool compared to resident responses to these two single-item questions is displayed in Figures 1 and 2. The single-item measure EE1 correlates with the EE(−EE1) subscale, and DP1 correlates with the DP(−DP1) subscale with Spearman’s rho of 0.81 (95% CI, 0.79–0.83) and 0.73 (95% CI, 0.70–0.75), respectively. Additional Spearman’s correlation data, comparing the primary and alternative definitions of burnout using the full MBI-HSS with single-item and subscale scores are reported in Table 2. Test characteristics for the 2-Question Summative Score (EE1+DP1) using different cutoff scores are reported in Table 3. The receiver operating characteristic (ROC) curve for primary, more inclusive, and more restrictive definitions of burnout based on the 2-Question Summative Score using different cutoffs is displayed in Figure 3. Using the primary definition of burnout, a summative score >3 demonstrated a sensitivity and specificity of 93.6% and 73.0%, respectively, compared to the full MBI-HSS. Applying this cutoff score of >3, 1183 of 1522 (77.7%) of residents would have been identified as burned out based on the responses from our original survey.

## DISCUSSION

In this study, we propose a rapid screen of burnout in the EM resident population, characterized as a 2-Question Summative Score based on self-reported frequency of emotional exhaustion and depersonalization. This simplified 2-Question Summative Score consists of two nested questions (EE1, DP1) in the MBI-HSS. A cutoff score >3 correlates best with the primary definition of burnout and the full MBI-HSS based on Spearman and ROC calculations (Table 2, Figure 3). A score of >3 can be obtained, for instance, if a resident reports feeling either burned out from work (EE1) or becoming more callous toward people since taking the job (DP1) at least once per week. Alternatively, burned-out residents would also be identified if they experienced both of these feelings but less frequently at once per month (e.g., each with a score of 2). This cutoff score demonstrates the best test characteristics compared to other cutoffs to the full MBI-HSS with a sensitivity, specificity, positive predictive value, and negative predictive value of 93.6%, 73.0%, 91.7%, and 78.2%, respectively, using the primary definition of burnout (Table 3). A cutoff with a high sensitivity was chosen because of the intent to use the summative score as a screening tool for burnout.

While other studies have examined the utility of abbreviated burnout measures in various physician and healthcare worker populations,^12^,^15^–^17^ to our knowledge this is the first study to determine the validity of an abbreviated, summative two-item burnout screening approach in the EM resident population. Among survey respondents, 77.7% of residents were identified as burned out by the 2-Question Summative Score, based on the single-item EE1 or DP1 scores. This is comparable to our previous study finding of a 76.1% burnout rate among EM residents using the full MBI-HSS.^9^

While prior studies report performance measures of single-item questions with their respective subscales in heterogeneous and non-EM populations,^12^,^15^–^17^ we initially hypothesized that such performance characteristics may be different in our population of EM-only residents. For instance, EM residents had shown a much higher prevalence of depersonalization (72.5%) compared to other resident burnout studies.^18^–^21^ However, our correlation values of 0.81 and 0.73 align with prior literature comparing EE1 and DP1 with full EE(−EE1) and DP(−DP1) subscales.^10^,^12^

It is important to acknowledge that there are numerous definitions of burnout as described in previous literature. For the purposes of this study, we chose a primary definition of burnout consistent with the original publication to determine the correlation of the 2-Question Summative Score with the full 22-item MBI instrument. However, we chose to also include analyses using more inclusive and more restrictive definitions of burnout to determine whether a correlation could also be demonstrated using existing alternative definitions. For both the primary and more inclusive burnout definitions, a 2-Question Summative Score >3 demonstrated adequate test characteristics with high sensitivities (Figure 3), suggesting that this cutoff may be applicable across either definition of burnout using the MBI-HSS tool. For the more restrictive definition of burnout, higher score cutoffs seem to demonstrate better agreement with the definition. Thus, stakeholders can apply different cutoffs based on their desire to identify burned out residents with a more inclusive or restrictive lens.

The 2-Question Summative Score is not meant to provide a comprehensive assessment of burnout and should not be considered a replacement for the full 22-item MBI instrument. Burnout is such a multidimensional phenomenon that two questions alone likely will not detect subtle differences and trends. Rather, this abbreviated score provides a reasonable alternative screening tool, supported by adequate correlative performance characteristics, to be used when the full tool is not available or not feasible to distribute.

## LIMITATIONS

Our study has limitations with respect to generalizability and nonresponse bias given the original survey methodology, which were addressed in the original publication.^9^ While prior publications studying the utility of a 2-item burnout screen obtained aggregate data from medical students, internal medicine residents, and practicing surgeons^10^ and pediatric residents,^12^ our study focuses on EM residents. Our results may not be generalizable outside the EM resident population. Specific analyses of subgroups (eg, male vs female, geographic region) with respect to the correlation of the 2-Question Summative Scale to the full MBI-HSS tool were not repeated as they were not found to have significant differences in the original publication.

Burnout is a multidimensional construct; simplifying the MBI into an abbreviated 2-question survey may miss the more nuanced and early characteristics of burnout among physicians, which would be captured using the full 22-item tool. Additionally, the 2-Question Summative Score is a tool limited by self-reporting bias and does not capture longitudinal facets of burnout.^22^

## CONCLUSION

In summary, with its brevity and ease of administration, the 2-Question Summative Score instrument has the ability to identify at-risk EM residents beginning to show signs of burnout. This simplified screening tool, which uses two MBI-HSS questions, has the potential to result in more widespread, consistent, and longitudinal monitoring of EM resident burnout on a local, regional, and national level by asking residents how often they feel burned out from work and how often they feel that have become more callous toward people since taking the job. This aligns with the 2017 Accreditation Council for Graduate Medical Education Common Program Requirements mandate focusing on improved resident well being and wellness education across health profession specialties.^23^ While tracking early burnout trends may help program leadership to implement early individual interventions, it is our hope that national organizations also use these trends to implement systemwide infrastructure and operational changes.^24^–^29^

## Figures and Tables

**Table 1:** Frequency of responses to the single-item questions “I feel burned out from my work” (EE1) and “I have become more callous toward people since I took this job” (DP1).

MBI-HSS Survey Response (Score)	EE1 Frequency (%)	DP1 Frequency (%)
Never (0)	81 (5.3)	124 (8.1)
A few times a year or less (1)	281 (18.5)	222 (14.6)
Once a month or less (2)	279 (18.3)	209 (13.7)
A few times a month (3)	318 (20.9)	255 (16.8)
Once a week (4)	257 (16.9)	289 (19.0)
A few times a week (5)	212 (13.9)	267 (17.5)
Every day (6)	94 (6.2)	156 (10.2)

*MBI-HSS*, Maslach Burnout Inventory-Human Services Survey; *EE*, emotional exhaustion; *DP*, depersonalization.

**Table 2:** Spearman’s rho correlation (95% confidence intervals) of MBI-HSS single-item measures and subscales compared to the primary, more inclusive, and more restrictive definitions of burnout from the 2017 Emergency Medicine Resident Wellness Survey.

MBI-HSS Items and Subscales	Primary definition	More inclusive definition	More restrictive definition
EE1	0.49 (0.45–0.53)	0.43 (0.39–0.46)	0.43 (0.40–0.47)
DP1	0.63 (0.60–0.66)	0.55 (0.52–0.58)	0.34 (0.30–0.38)
EE(−EE1)	0.59 (0.56–0.62)	0.51 (0.48–0.55)	0.48 (0.45–0.51)
DP(−DP1)	0.69 (0.66–0.71)	0.60 (0.57–0.63)	0.36 (0.32–0.40)
EE1+DP1	0.65 (0.62–0.68)	0.57 (0.53–0.60)	0.44 (0.41–0.48)

“I feel burned out from my work” (EE1). “I have become more callous toward people since I took this job” (DP1).

*MBI-HSS*, Maslach Burnout Inventory-Human Services Survey; *EE*, emotional exhaustion; *DP*, depersonalization.

**Table 3:** Sensitivity, specificity, positive predictive value (PPV), and negative predictive value (NPV) the 2-Question Summative Score compared to the primary, more inclusive, and more restrictive definitions of burnout by the full Maslach Burnout Inventory-Human Services Survey.

Score	Test characteristic	Primary definition	More inclusive definition	More restrictive definition
>3	Sensitivity	93.6	90.0	100.0
	Specificity	73.0	74.2	27.2
	PPV	91.7	93.7	23.4
	NPV	78.2	63.7	100.0
>4	Sensitivity	85.9	81.4	98.2
	Specificity	87.3	86.6	38.2
	PPV	95.6	96.3	26.1
	NPV	65.9	52.4	99.0
>5	Sensitivity	74.7	70.5	95.3
	Specificity	95.0	94.5	50.2
	PPV	98.0	98.2	29.9
	NPV	54.1	43.1	98.0
>6	Sensitivity	60.1	56.5	87.0
	Specificity	99.7	99.7	63.4
	PPV	99.9	99.9	34.6
	NPV	43.9	35.2	95.6

*PPV*, postivie predictive value; *NPV*, negative predictive value.
